# Intraoperative Strahlentherapie in der Abdominalchirurgie – eigene Erfahrungen

**DOI:** 10.1007/s00104-020-01165-z

**Published:** 2020-04-08

**Authors:** Katharina Joechle, Eleni Gkika, Anca-Ligia Grosu, Ulrich T. Hopt, Hannes P. Neeff, Stefan Fichtner-Feigl, Sven A. Lang

**Affiliations:** 1 Klinik für Allgemein- und Viszeralchirurgie, Universitätsklinikum Freiburg, Hugstetter Str. 55, 79106 Freiburg, Deutschland; 2 Klinik für Strahlenheilkunde, Universitätsklinikum Freiburg, Freiburg, Deutschland

**Keywords:** Intraoperative Radiotherapie, Rektumkarzinom, Sarkom, Analkarzinom, Lokalrezidiv, Intraoperative radiotherapy, Rectal carcinoma, Sarcoma, Anal carcinoma, Local tumor recurrence

## Abstract

**Hintergrund:**

Die intraoperative Radiotherapie (IORT) kann bei lokal weit fortgeschrittenen Tumoren und zu erwartender bzw. nicht vermeidbarer R1-Situation ergänzend zur chirurgischen Resektion eingesetzt werden. Ziel ist eine verbesserte lokale Tumorkontrolle und damit ein besseres Langzeitüberleben. Indikationen sind sowohl primäre intraabdominelle und retroperitoneale Tumoren als auch Rezidivtumoren. Im Rahmen der vorliegenden Arbeit werden die eigenen Erfahrungen mit der Durchführung einer IORT bei viszeralchirurgischen Resektionen zusammengefasst.

**Methodik:**

Patienten, die von Januar 2008 bis Dezember 2018 eine IORT kombiniert mit abdomineller Tumorresektion in der Klinik für Allgemein- und Viszeralchirurgie des Universitätsklinikums Freiburgs erhalten hatten, wurden in diese Arbeit eingeschlossen und hinsichtlich Kurz- und Langzeitergebnisse evaluiert.

**Ergebnisse:**

Die häufigste Indikation zur Durchführung einer IORT stellten Sarkome gefolgt von Rektum- und Analkarzinomen dar. Die mediane angewandte Strahlendosis der IORT betrug 15 Gy (8–19 Gy). Bei einem medianen „comprehensive complication index“ (CCI) von 11,9 traten bei 24 % der Patienten Komplikationen (Dindo-Clavien ≥ °III) auf. Die 90-Tage-Mortalität betrug 0 %. Besonders für Analkarzinomrezidive war die lokale Kontrolle nach einem Jahr trotz R0-Resektion unzureichend.

**Schlussfolgerung:**

In unserem Patientenkollektiv war die IORT mit vertretbarer Morbidität einsetzbar. Dennoch sind Indikationsstellung und Patientenselektion kritische Punkte für die Durchführung der Behandlung. Der Effekt der IORT zur Verbesserung der lokalen Kontrolle und damit auch des Langzeitüberlebens sollte in weiteren Studien evaluiert werden.

## Abstract (en)

**Background:**

Intraoperative radiotherapy (IORT) can be applied for locally advanced tumors and expected or unavoidable R1 situations combined with surgical resection. The aim is to improve local tumor control and long-term survival. The indications are primary and recurrent intra-abdominal and retroperitoneal tumors. This study aimed to evaluate own data and experiences with IORT combined with surgical visceral resection.

**Methods:**

Patients who underwent IORT combined with abdominal tumor resection in the Department of General and Visceral Surgery at the University Medical Center Freiburg between January 2008 and December 2018 were included in this study. The results were retrospectively evaluated regarding short-term and long-term outcomes.

**Results:**

The most frequent indications for IORT were sarcoma followed by rectal and anal cancers. The median IORT dose used was 15 Gy (range 8–19 Gy). With a median comprehensive complication index (CCI) of 11.9, complications occurred in 24% of patients (Dindo-Clavien ≥ °III). The 90-day mortality was 0%. Especially in recurrent anal cancer the local control after 1 year was insufficient despite R0 resection.

**Conclusion:**

In this cohort of patients IORT could be applied with acceptable morbidity. Nevertheless, the indications and patient selection are critical factors for carrying out the treatment. The effect of IORT to improve local tumor control and long-term survival should be evaluated in further studies.

## Hintergrund

Die intraoperative Radiotherapie (IORT) gilt als effektive Option zur Verbesserung der lokalen Tumorkontrolle und damit auch des Gesamtüberlebens bei verschiedenen Tumorentitäten [15]. Als vorteilhaft für die IORT wird die hohe Präzision durch chirurgische Exposition des Tumors bzw. des Tumorbetts sowie eine Eskalation der Strahlendosis bei gleichzeitigem Schutz gesunden Gewebes als dem dosislimitierenden Faktor bei der perkutanen Bestrahlung angesehen [13, 15]. Die Evidenz bezüglich der adäquaten Strahlendosis und der Relation zwischen Dosis und zu erzielendem Effekt ist bislang jedoch unzureichend, was den Nachweis eines therapeutischen Benefits für einzelne Tumorentitäten erschwert [8]. Dennoch kann die IORT sowohl bei primären intraabdominellen und retroperitonealen Tumoren als auch bei Tumorrezidiven ergänzend zur chirurgischen Resektion Erfolg versprechend eingesetzt werden [15].

In der Viszeralchirurgie stellen Sarkome die Hauptindikation zur Durchführung der IORT im Rahmen multimodaler Therapiekonzepte dar [15, 21]. Zudem kann die IORT als individuelle Option bei lokal weit fortgeschrittenen Rektumkarzinomen oder Rektumkarzinomrezidiven erfolgreich angewandt werden [15, 21]. Verschiedene Fallserien berichten auch über den Einsatz bei gynäkologischen Rezidivtumoren, wo mittels IORT teils gute lokale Tumorkontrolle erreicht werden konnte [5, 16, 22, 24]. Für weitere viszerale Tumorentitäten wie Pankreas- oder Magenkarzinome sowie hepatobiliäre Tumoren ist die Datenlage zur Rolle der IORT bislang limitiert. Obwohl die IORT bei einigen der aufgeführten Indikationen durchaus im klinischen Alltag etabliert ist, stellt die Patientenselektion einen entscheidenden Faktor für deren Erfolg dar [15]. Entsprechend sollte die Behandlung nicht mit einer gravierenden Zunahme der Morbidität und Mortalität einhergehen.

Das Ziel dieser Arbeit war die Erfassung der eigenen Daten zur Durchführung einer IORT kombiniert mit chirurgischer Resektion bezüglich der perioperativen und, sofern vorhanden, onkologischen Ergebnisse.

## Material und Methoden

### Studienpopulation

Patienten, welche eine intraoperative Strahlentherapie im Rahmen der Resektion eines intraabdominellen oder retroperitonealen Tumors bzw. einer Rezidivresektion in der Klinik für Allgemein- und Viszeralchirurgie des Universitätsklinikums Freiburg von Januar 2008 bis Dezember 2018 erhielten, wurden nach Bewilligung durch die Ethikkommission der Albert-Ludwigs-Universität Freiburg (220/19) in diese Arbeit eingeschlossen.

### Perioperatives Management

Die Entscheidung, die chirurgische Resektion um eine intraoperative Strahlentherapie potenziell zu ergänzen, wurde während der präoperativen Patientenevaluation in enger Zusammenarbeit der Kliniken für Allgemein- und Viszeralchirurgie und Strahlentherapie bei erwarteter R1-Resektion bzw. zu erwartendem sehr knappem Resektionsrand getroffen und im interdisziplinären Tumorboard diskutiert.

Die Kontrolluntersuchungen umfassten die klinische, laborchemische und bildmorphologische Untersuchung im Abstand von 3 bis 4 Monaten.

### IORT

Der definitive Entschluss zur IORT erfolgte intraoperativ bei vorliegender R1-Situation bzw. sehr knappem Resektionsrand. Je nach Anforderung an das Strahlenfeld werden am Universitätsklinikum Freiburg das Interbeam- oder das Mobetron-Gerät verwendet. Ersteres appliziert niederenergetische Röntgenstrahlen mit hoher biologischer Wirksamkeit im Nahbereich, während das Mobetron-Gerät Elektronenstrahlung verwendet. Durch deren geringe Eindringtiefe werden besonders tiefer liegende, gesunde Organe geschont.

### Datenerhebung

Folgende Patientendaten wurden retrospektiv erhoben: Geschlecht, Alter, Art der Tumorerkrankung, Rezidivstatus, TNM-Klassifikation, Art der Operation, intraoperative Parameter (Operationszeit, Blutverlust), Dosis der intraoperativen Strahlentherapie, Resektionsstatus, perioperative Parameter (Komplikationen, „comprehensive complication index“ [CCI], Dauer des Krankenhausaufenthalts, ungeplante Wiederaufnahme, 90-Tage-Mortalität), neoadjuvante bzw. adjuvante Chemo- bzw. Radiotherapie, Überlebens- und Rezidivraten. Postoperative Komplikationen wurden nach Dindo-Clavien [25] klassifiziert und Komplikationen °IIIb oder höher als schwere Komplikation gewertet. Aufgrund des heterogenen Patientenkollektivs und der z. T. sehr unterschiedlichen Nachbeobachtungszeiträume wurden die Überlebensdaten nur bis zu 12 Monaten postoperativ erfasst.

### Statistische Auswertung

Die statistische Analyse der Daten erfolgte mittels SPSS (Version 22.0; IBM, Armonk, New York, USA). Stetige Variablen sind als Median und Spannweite angegeben, kategoriale Daten als Anzahl und Prozent. Überlebensdaten wurden vom Tag der Resektion mit IORT berechnet und mittels Kaplan-Meier-Methode abgeschätzt.

## Ergebnisse

### Patientencharakteristika

Zwischen 2008 und 2018 erhielten 58 Patienten eine IORT kombiniert mit chirurgischer Resektion. Die häufigste Indikation stellten Sarkome mit 26 Patienten (8 primäre Sarkome, 18 Sarkomrezidive) sowie Rektumkarzinome mit 21 Patienten (7 lokal weit fortgeschrittene primäre Rektumkarzinome, 14 Rektumkarzinomrezidive) dar. Zudem hatten 4 Patienten Analkarzinomrezidive, 6 Patientinnen gynäkologische Rezidivtumoren (Endometriumkarzinom, Zervixkarzinom, Ovarialkarzinom und Vaginalkarzinom) und 1 Patient ein retroperitoneales Rezidiv eines Pankreaskarzinoms. Patienten, die eine IORT kombiniert mit chirurgischer Resektion erhielten, waren im Median 66 (23–79) Jahre alt und in der Mehrzahl männlich (34/58; 59 %). Die mediane angewandte Strahlendosis der IORT lag bei 15 Gy (8–19 Gy). Die Ergebnisse sind in Tab. 1 zusammengefasst.

**Table 1:** 

		Patientenkollektiv *n* = 58 (100 %)
Geschlecht, *n* (%)	Männlich	34 (59)
	Weiblich	24 (41)
Alter in Jahren, Median (Spannweite)		65,5 (23–79)
Indikationen, *n* (%)	Primäre Sarkome	8 (14)
	Sarkomrezidive	18 (31)
	Lokal fortgeschrittene Rektumkarzinome	7 (12)
	Rektumkarzinomrezidive	14 (24)
	Analkarzinomrezidive	4 (7)
	Andere Karzinome	7 (12)

### Perioperative Behandlung

Eine neoadjuvante Behandlung wurde bei 13 Patienten (22 %) durchgeführt. Acht Patienten (14 %) erhielten eine kombinierte Radiochemotherapie, 2 Patienten eine neoadjuvante Chemotherapie und 3 Patienten eine neoadjuvante Radiatio. Eine adjuvante Therapiestrategie erhielten insgesamt 18 Patienten, wobei hierzu nur Daten von 50 der 58 Patienten vorlagen. Sechs Patienten (12 %) wurden einer adjuvanten Radiatio, 10 Patienten (20 %) einer adjuvanten Chemotherapie und 2 Patienten (3 %) einer adjuvanten Radiochemotherapie zugeführt. Die Ergebnisse sind in Tab. 2 zusammengefasst.

**Table 2:** 

	Gesamtes Kollektiv *n* = 58 (100 %)	Primäre Sarkome *n* = 8 (14 %)	Sarkomrezidive *n* = 18 (31 %)	Lokal fortgeschrittenes Rektum-Ca *n* = 7 (12 %)	Rektum-Ca‐Rezidive *n* = 14 (24 %)	Anal-Ca‐Rezidive *n* = 4 (7 %)	Andere Karzinome *n* = 7 (12 %)
Radiatio im Krankheitsverlauf (bei Rezidivtumoren), *n* (%)	27 (47)	–	11 (61)	–	7 (50)	4 (100)	5 (71)
Neoadjuvante Radiatio, *n* (%)	3 (5)	2 (25)	1 (6)	0 (0)	0 (0)	0 (0)	0 (0)
Neoadjuvante Chemotherapie, *n* (%)	2 (3)	0 (0)	0 (0)	0 (0)	1 (7)	0 (0)	1 (14)
Neoadjuvante Radiochemotherapie, *n* (%)	8 (14)	0 (0)	0 (0)	7 (100)	1 (7)	0 (0)	0 (0)
Adjuvante Radiatio^a^, *n* (%)	6 (12)	1 (14)	4 (25)	0 (0)	1 (7)	0 (0)	0 (0)
Adjuvante Chemotherapie^a^, *n* (%)	10 (20)	1 (14)	1 (6)	4 (57)	4 (33)	1 (25)	0 (0)
Adjuvante Radiochemotherapie^a^, *n* (%)	2 (3)	0 (0)	0 (0)	0 (0)	1 (7)	0 (0)	1 (14)

^a^Daten verfügbar für 50 Patienten

### Resektionsausmaß

Das Resektionsausmaß bei Patienten mit Sarkom (*n* = 8) bzw. Sarkomrezidiv (*n* = 18) umfasste eine alleinige Tumorexstirpation bei 50 % der Patienten (4/8) mit primärem Sarkom und 44 % der Patienten (8/18) mit Sarkomrezidiv. Bei den verbleibenden Sarkompatienten (primär 4/8, 50 %; Rezidiv 10/18, 56 %) mussten eine oder mehrere begleitende Organresektionen vorgenommen werden (Nephrektomie, Adrenalektomie, Splenektomie, Hemikolektomie, Gastrektomie, Dünndarmteilresektion, Pankreatoduodenektomie, atypische Leberresektion, Orchiektomie, Exenteratio plevis).

Patienten mit lokal weit fortgeschrittenem Rektumkarzinom (*n* = 7) wurden im präoperativen, klinischen Staging als cT4 (5/7; 71 %) bzw. cT3 (2/7; 29 %) und cN2 (5/7; 71 %) bzw. cN1 (2/7; 29 %) eingestuft und konnten in 71 % der Fälle (5/7 Patienten) mittels tiefer anteriorer Rektumresektion (hiervon bei jeweils einem Patienten [14 %] kombiniert mit vorderer Exenteratio bzw. Hemikolektomie links) reseziert werden. Bei 29 % der Patienten (2/7) musste eine Rektumexstirpation erfolgen. Bei Patienten mit Rektumkarzinomrezidiv (*n* = 14) gelang eine alleinige Tumorexzision des Lokalrezidivs in 43 % der Fälle (6/14). Bei 5/14 Patienten (36 %) war eine simultane Organresektion notwendig (Dünndarmteilresektion, Nephrektomie, Ureterresektion, Zystektomie), eine Rektumexstirpation erfolgte bei einem Patienten (7 %) und eine Exenteratio pelvis bei weiteren 2/14 Patienten (14 %).

Patienten mit Analkarzinomrezidiv erhielten in 50 % der Fälle (2/2) eine Exenteratio pelvis, bei je einem Patienten (25 %) erfolgte eine lokale Tumorexzision bzw. eine Tumorexzision mit Ovarektomie und Os-coccygis- und partieller Os-sacrum-Resektion. Bei den Patientinnen mit gynäkologischen Rezidivtumoren (*n* = 6) konnte bei 2/6 Patienten (33 %) eine lokale Tumorexzision erfolgen, bei 2 weiteren Patienten (33 %) war neben der Tumorexzision eine Dünndarmsegmentresektion bzw. ein Vena-cava-Ersatz notwendig. Die restlichen beiden Patienten erhielten eine Exenteratio pelvis. Der Patient mit lokalem Rezidiv eines Pankreaskarzinoms wurde einer lokalen Tumorresektion mit Lymphadenektomie zugeführt.

### Postoperative Morbidität

**Figure 1:**
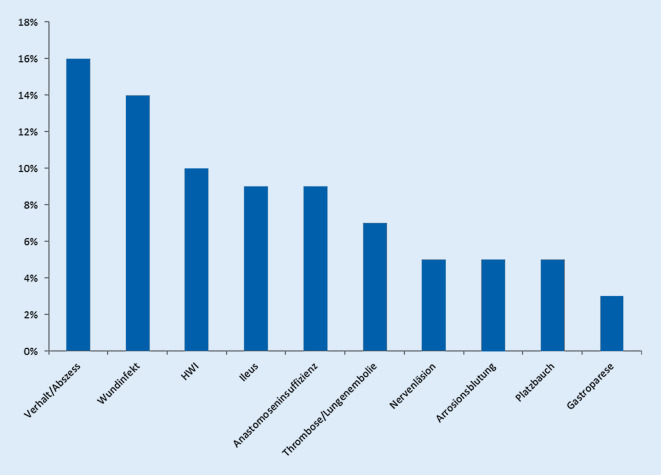

Die postoperative Gesamtkomplikationsrate betrug 59 % (34/58 Patienten). Dabei traten Abszesse/Verhalte und Wundheilungsstörungen mit 16 % und 14 % als häufigste Komplikationen auf. Weitere häufig auftretende Komplikationen waren Harnwegsinfekte (10 %), Ileus und Anastomoseninsuffizienzen (je 9 %) sowie Thrombosen oder Lungenembolien (7 %) (Abb. 1). Eine Nervenläsion als oftmals IORT-assoziierte Komplikation trat in unserem Kollektiv bei 3 Patienten (5 %) auf. Hiervon erhielten 2 Patienten eine lokale Tumorexstirpation mit Lymphadenektomie bei primärem Sarkom, während der 3. Patient aufgrund eines lokal fortgeschrittenen Rektumkarzinoms eine tiefe anteriore Rektumresektion erhalten hatte. In Folge postoperativer Komplikationen musste bei 8/58 Patienten (14 %) eine Intervention (Komplikation °IIIa nach Dindo-Clavien) erfolgen. Schwere Komplikationen im Sinne einer Komplikation nach Dindo-Clavien ≥ °IIIb traten bei 14/58 Patienten (24 %) auf und waren besonders für Patienten mit Rezidivresektionen bei Analkarzinom (2/4, 50 %) und Rektumkarzinom (5/14, 36 %) erhöht. Einschränkend muss hier sicherlich die geringe Patientenzahl insbesondere bei Patienten mit Analkarzinomrezidiv gesehen werden. Die mediane Aufenthaltsdauer im Krankenhaus betrug 13 Tage (6–136). Der mediane CCI lag bei 11,9 (0–100). Die 90-Tage-Mortalität betrug 0 %. Die Ergebnisse sind in Tab. 3 zusammengefasst.

**Table 3:** 

	Gesamtes Kollektiv *n* = 58 (100 %)	Primäre Sarkome *n* = 8 (14 %)	Sarkom Rezidive *n* = 18 (31 %)	Lokal fortgeschrittenes Rektum-Ca *n* = 7 (12 %)	Rektum-Ca‐Rezidive *n* = 14 (24 %)	Anal-Ca‐Rezidive *n* = 4 (7 %)	Andere Karzinome *n* = 7 (12 %)
Dosis IORT, Gy, Median (Spannweite)	15 (8–19)	13,5 (12–15)	15 (8–18)	15 (12–15)	15 (12–19)	12 (12–13)	14 (12–18)
Operationsdauer, min, Median (Spannweite)	408 (179–911)	317 (196–428)	347 (179–747)	490 (385–561)	394 (213–755)	627 (409–911)	369 (202–780)
Blutverlust, ml, Median (Spannweite)	500 (30–3400)	425 (200–1000)	400 (50–3000)	500 (300–1000)	600 (30–1900)	1000 (700–1000)	600 (100–3400)
Postoperative Komplikation, *n* (%)	34 (59)	3 (38)	7 (39)	6 (86)	10 (71)	3 (75)	5 (71)
Dindo-Clavien ≥ IIIb, *n* (%)	14 (24)	1 (13)	3 (17)	1 (14)	5 (36)	2 (50)	2 (29)
CCI, Median (Spannweite)	11,9 (0–100)	11,9 (0–47,7)	8,7 (0–63)	20,9 (0–52,1)	22,3 (0–60,4)	27,9 (0–34,8)	26, (0–100)
Krankenhausaufenthalt, Tage, Median (Spannweite)	13 (6–136)	13 (6–28)	10 (6–87)	13 (7–45)	18 (7–64)	22 (13–34)	20 (8–136)
Ungeplante Wiederaufnahme innerhalb von 45 Tagen, *n* (%)	11 (19)	0 (0)	3 (17)	1 (14)	4 (29)	2 (50)	1 (14)
90-Tage-Mortalität, *n* (%)	0 (0)	0 (0)	0 (0)	0 (0)	0 (0)	0 (0)	0 (0)

*CCI* Comprehensive Complication Index, *IORT* intraoperative Strahlentherapie

### Tumorkontrolle

Bei 33 Patienten (57 %) des Gesamtkollektivs konnte eine R0-Situation erreicht werden. Der mediane Nachbeobachtungszeitraum lag bei 16,5 Monaten. Aufgrund des kurzen medianen Nachbeobachtungszeitraums sind die Überlebensdaten im Folgenden nur für 12 Monate postoperativ angegeben (Tab. 4). Für die Patientinnen mit gynäkologischen Rezidivtumoren (6/58, 10 %) sowie für den Patienten mit Lokalrezidiv eines Pankreaskarzinoms wird aufgrund der geringen Anzahl der einzelnen Tumorentitäten sowie der Heterogenität dieser Patienten auf eine Analyse der Überlebensdaten verzichtet. Die Ergebnisse für Patienten mit primärem Sarkom, Sarkomrezidiv, primärem Rektumkarzinom, Rektumkarzinomrezidiv und Analkarzinomrezidiv sind in Tab. 4 zusammengefasst.

**Table 4:** 

	Mediane Nachbeobachtungszeit	Resektionsstatus	Lokalrezidiv	Lokalrezidiv + Fernmetastasierung	Fernmetastasierung	Tumorfrei	Lost-to‐Follow-up
Primäre Sarkome *n* =8	9 Monate (1–86)	R0 *n* = 6 (75 %)	1	0	0	3	2
		R1 *n* = 2 (25 %)	1	0	0	0	1
Sarkomrezidive *n* = 18	14 Monate (1–46)	R0 *n* = 9 (50 %)	0	3^a^	1	2	3
		R1 *n* = 9 (50 %)	2^a^	1	3	2	1
Lokal fortgeschrittenes Rektum-Ca *n* =7	16 Monate (2–54)	R0 *n* = 7 (100 %)	0	1	2	2^a^	2
Rektum-Ca-Rezidive *n* = 14	11 Monate (1–56)	R0 *n* = 4 (29 %)	0	0	0	3	1
		R1 *n* = 10 (71 %)	1	2	3	2	2
Anal-Ca-Rezidive *n* = 4	23 Monate (4–82)	R0 *n* = 4 (100 %)	0	2	1^a^	1	0

^a^Jeweils 1 Patient innerhalb von 12 Monaten postoperativ verstorben

## Diskussion

Mit der vorliegenden Arbeit berichten wir über eigene Erfahrungen und Ergebnisse der IORT, die im Rahmen viszeralchirurgischer Operationen durchgeführt wurde. In unserem Patientenkollektiv waren die Hauptindikationen Sarkome und Rektumkarzinome, was auch der vorhandenen Literatur entspricht [15, 21]. Trotz der hohen Anzahl von Patienten mit strahlentherapeutischer Vorbehandlung (im Krankheitsverlauf oder neoadjuvant; 40/58; 69 %) stellte die IORT eine gute Möglichkeit dar, diese Patienten erneut effektiv strahlentherapeutisch zu behandeln. Dabei wurde eine mediane Strahlendosis von 15 Gy (8–19 Gy) angewandt. Die Rate an schwerwiegenden Komplikationen betrug 24 %, wobei Abszesse/Verhalte und Wundinfekte mit 16 % und 14 % die häufigsten Komplikationen darstellten. Kein Patient verstarb innerhalb von 90 Tagen nach der Operation/IORT. Der mediane Nachbeobachtungszeitraum der eingeschlossenen Patienten lag bei nur 16,5 Monaten.

Aufgrund der Heterogenität unserer Patientenkohorte sowie des limitierten Nachbeobachtungszeitraums lassen sich die mittelfristigen Überlebensdaten auch nur schwer mit der vorhandenen Literatur vergleichen. Dennoch scheinen diese beispielsweise für Rektumkarzinomrezidive vergleichbar zu sein. Dresen et al. [4] beschreiben eine 1‑Jahres-Lokalrezidivrate von 15 %, während diese in unserem Kollektiv 21 % (3/14 Patienten; 1/14 Patient mit alleinigem Lokalrezidiv sowie 2/14 Patienten mit Lokalrezidiv und Fernmetastasierung) betrug.

Welche Patienten von einer IORT profitieren können, ist nach Studienlage bisher nicht abschließend geklärt. Jedoch wird besonders für Patienten mit primärem, lokal fortgeschrittenem Rektumkarzinom die IORT nur bei positiven Resektionsrändern ohne Möglichkeit der funktionserhaltenden Nachresektion empfohlen. So konnten nur bei R1-Resektion übereinstimmend sowohl verbesserte Lokalrezidiv- als auch Gesamtüberlebensraten mittels IORT verzeichnet werden [7, 10, 11, 20]. Obwohl die in unserer Serie eingeschlossenen Patienten mit primärem Rektumkarzinom eine IORT aufgrund einer zu erwartenden R1-Situation oder mutmaßlich knapper Resektionsränder erhielten, wurde in allen Fällen eine R0-Resektion erreicht. Vor diesem Hintergrund ist die geringe Lokalrezidivrate, die bei dieser Indikation beobachtet wurde, sicher nicht klar als Erfolg der IORT anzusehen, sondern muss im Gesamtkontext betrachtet werden. Der Resektionsstatus gilt auch für Rektumkarzinomrezidive und Sarkome als wichtiger prognostischer Faktor für die Entwicklung eines lokalen (Re‑)Rezidivs, sodass die R1-Situation eine Indikation zur Durchführung einer IORT darstellt. Im Rahmen dessen ist beispielsweise auch die hohe R1-Resektionsrate von 71 % für Rektumkarzinomrezidive zu erklären. In unserem Kollektiv wiesen nur 3/10 Patienten nach 12 Monaten ein erneutes Lokalrezidiv auf. Ein weiterer Faktor, welcher die lokale Tumorkontrolle für Rektumkarzinomrezidive und Sarkome zu beeinflussen scheint, ist die Dosisaufsättigung mittels neoadjuvanter oder adjuvanter Radiatio. Auch wenn hierzu aus den eigenen Daten eine Aussage nur schwierig abzuleiten ist, so konnten Studien für Patienten mit Rektumkarzinomrezidiven, welche vor Resektion mit IORT eine perkutane Bestrahlung erhalten hatten, verbesserte Lokalrezidivraten zeigen [1, 9, 18]. Dieser Effekt trat besonders bei R1-Situation und kurzer Wartezeit von <7 Wochen zwischen perkutaner Bestrahlung und Resektion mit IORT auf [9]. Auch für Patienten mit primären und rezidivierenden Sarkomen >5 cm, welche vor Resektion mit IORT mittels perkutaner Radiatio behandelt wurden, konnte eine sehr gute 5‑Jahres-Lokalrezidivrate von 28 % erreicht werden [19]. Im Gegensatz dazu scheint die Indikationsstellung der IORT bei Patienten mit Analkarzinomrezidiven schwierig und die Ergänzung des radikalen chirurgischen Vorgehens um eine IORT positive Resektionsränder nicht zu kompensieren [3]. Allerdings ist die Datenlage bezüglich der IORT für Analkarzinomrezidive generell limitiert. So existiert diesbezüglich nur eine kleine Fallserie von 14 Patienten, die die Durchführung einer IORT verbunden mit „Salvage“-Resektion untersuchte [3]. Bereits nach im Median 8 Monaten entwickelten 11/14 Patienten ein Rerezidiv. Die Autoren schlussfolgerten, dass auch mittels radikalem chirurgischem Vorgehen kombiniert mit IORT keine suffiziente lokale Tumorkontrolle erreicht werden könne [3]. Dies lässt sich trotz unseres geringen Patientenkollektivs von nur 4 Patienten mit Analkarzinomrezidiven auf die eigenen Daten übertragen; bereits nach 4 Monaten (Median) wiesen die Hälfte der Patienten ein erneutes Lokalrezidiv inklusive Fernmetastasierung auf und nach 8 Monaten (Median) zeigten alle Patienten eine Fernmetastasierung. Diese ungenügende lokale Tumorkontrolle zeigte sich trotz R0-Resektion bei allen Patienten.

Aus diesen Daten und aufgeführten Studien lässt sich auf die Wichtigkeit der Patientenselektion zur Durchführung einer IORT schließen. Während zum einen die Tumorentität selbst ein wichtiger Faktor zu sein scheint und Patienten mit Analkarzinomrezidiven möglicherweise nur wenig von einer IORT profitieren, sind Rektumkarzinome und Sarkome häufig beschriebene Indikationen. Hierfür ist die IORT aktiv im klinischen Alltag etabliert, obwohl die Evidenzlage zur Effektivität der IORT auch für diese beiden Tumorentitäten nur gering ist. Bei den meisten Studien handelt es sich um Erfahrungen aus einzelnen Zentren, teils mit sehr kleinen Patientenkollektiven. Prospektive, randomisiert-kontrollierte Studien fehlen bisher. Zum anderen stellt das Risiko, ein Lokalrezidiv zu entwickeln, einen guten Selektionsmarker zur Durchführung einer IORT dar und ist besonders für Patientinnen mit Mammakarzinom bereits gut definiert. Entsprechend müssen nicht nur die Histologie und die bisherigen Behandlungsstrategien und Tumorrezidive des Patienten, sondern auch die zu erwartenden Resektionsränder berücksichtigt werden. Somit stellt beispielsweise die R1-Resektion bei Rektumkarzinomen eine gute Indikation zur Durchführung einer IORT dar.

Unabhängig von der jeweiligen Indikation und Tumorentität erscheint die Morbiditätsrate von 59 % (34/58) in unserer Serie zunächst relativ hoch. Dabei trat eine schwerwiegende Komplikation bei 24 % der Patienten (14/58) auf. Diese Daten sind jedoch vergleichbar mit vorangegangen Arbeiten; so beschreiben beispielsweise Mirnezami et al. in einer Metaanalyse zur IORT bei Rektumkarzinomen eine Komplikationsrate von bis zu 59 % [12]. Ähnliche Ergebnisse berichten Roeder et al. mit 34 % schwerwiegenden Komplikationen nach IORT bei Sarkomen [17]. Auch im Vergleich der Komplikationsrate nach operativen Eingriffen mit und ohne IORT zeigt sich diese nicht signifikant erhöht: Beispielsweise beschreiben Dubois et al. [6] das Auftreten von Komplikationen in 29,6 % vs. 19,1 % (*p* = 0,15) der Fälle für Patienten mit lokal fortgeschrittenem Rektumkarzinom mit bzw. ohne IORT. Für lokal rezidivierende Rektumkarzinome mit und ohne IORT sind ebenfalls ähnliche Raten an schwerwiegenden Komplikationen beschrieben [23]. Als besonders häufige und relevante Komplikation nach IORT werden jedoch Wundheilungsstörungen genannt [3, 12, 15, 17]. Dies spiegelt sich auch in unseren Daten wider: Wundheilungsstörungen waren neben Verhalten/Abszessen mit jeweils 14 % bzw. 16 % die häufigsten Komplikationen. Während die hohe Rate an Verhalten/Abszessen möglicherweise auch als Folge des radikalen chirurgischen Vorgehens gewertet werden kann, ist die lokale Neuropathie sicherlich ein weitgehend IORT-assoziierter Faktor. Diese trat in unserer Serie in 5 % der Fälle auf, wovon 2 von 3 Patienten an einem primären Sarkom litten. Analysiert man Studien zur Neuropathie nach IORT bei Sarkomen, so sind vergleichbare Raten von bis zu 10 % beschrieben [2, 14]. Um die Morbiditätsraten in unserer Serie abschließend zu bewerten, muss gewiss beachtet werden, dass der Großteil der Patienten (39/58; 67 %) an einem Rezidivtumor litt und somit bereits ausgiebig voroperiert wurde und ggf. zusätzlich vorbestrahlt war, was das chirurgische Vorgehen wiederum erschwert und das Komplikationsprofil beeinflusst. Dabei ist die neoadjuvante Radiatio – abhängig von der applizierten Dosis – ein weiterer Faktor, welcher besonders das Auftreten von Wundinfektionen erhöht. Eine neoadjuvante Radiatio (inklusive Radiochemotherapie) erhielten in unserem Kollektiv 19 % der Patienten. Vor diesem Hintergrund lässt sich bei vergleichbaren Morbiditätsraten in der Literatur auf eine gute Machbarkeit der IORT schließen. Zudem spiegelt der mediane CCI von 11,9 und die 90-Tage-Mortalität von 0 % die Seltenheit relevanter Komplikationen wider.

Einige Limitationen müssen bezüglich dieser Arbeit genannt werden. Durch das retrospektive Design und die Evaluation einer selektiven Patientenkohorte an nur einer Institution besteht die Möglichkeit eines Selektionsbias. Patienten, die die IORT kombiniert mit radikaler chirurgischer Resektion erhielten, wiesen möglicherweise einen aggressiven Erkrankungsverlauf auf, sodass die Entscheidung zur Durchführung der IORT begünstigt wurde. Des Weiteren muss limitierend der kurze mediane Nachsorgezeitraum in unserem Kollektiv sowie die geringe Patientenanzahl in den einzelnen, teilweise heterogenen Gruppen genannt werden, der eine effektive Überlebensanalyse erschwert.

## Schlussfolgerung

Die IORT zeigte in unserem Patientenkollektiv eine gute Machbarkeit für alle Tumorentitäten. Um lokale Tumorkontrolle und Langzeitüberleben zu erreichen, ist die Indikationsstellung ein kritischer Punkt, der weitere Untersuchungen erfordert. Besonders für Analkarzinomrezidive war die lokale Kontrolle nach einem Jahr trotz R0-Resektion unzureichend.

## References

[1] Calvo FA, Sole CV, Alvarez De Sierra P (2013). Prognostic impact of external beam radiation therapy in patients treated with and without extended surgery and intraoperative electrons for locally recurrent rectal cancer: 16-year experience in a single institution. Int J Radiat Oncol Biol Phys.

[2] Calvo FA, Sole CV, Obregon R (2013). Intraoperative radiotherapy for the treatment of resectable locally advanced gastric adenocarcinoma: topography of locoregional recurrences and long-term outcomes. Clin Transl Oncol.

[3] Coquard R, Ayzac L, Gilly FN (1997). Intraoperative radiation therapy combined with limited lymph node resection in gastric cancer: an alternative to extended dissection?. Int J Radiat Oncol Biol Phys.

[4] Dresen RC, Gosens MJ, Martijn H (2008). Radical resection after IORT-containing multimodality treatment is the most important determinant for outcome in patients treated for locally recurrent rectal cancer. Ann Surg Oncol.

[5] Drognitz O, Henne K, Weissenberger C (2008). Long-term results after intraoperative radiation therapy for gastric cancer. Int J Radiat Oncol Biol Phys.

[6] Dubois JB, Bussieres E, Richaud P (2011). Intra-operative radiotherapy of rectal cancer: results of the French multi-institutional randomized study. Radiother Oncol.

[7] Ferenschild FT, Vermaas M, Nuyttens JJ (2006). Value of intraoperative radiotherapy in locally advanced rectal cancer. Dis Colon Rectum.

[8] Hensley FW (2017). Present state and issues in IORT physics. Radiat Oncol.

[9] Holman FA, Bosman SJ, Haddock MG (2017). Results of a pooled analysis of IOERT containing multimodality treatment for locally recurrent rectal cancer: results of 565 patients of two major treatment centres. Eur J Surg Oncol.

[10] Holman FA, Haddock MG, Gunderson LL (2016). Results of intraoperative electron beam radiotherapy containing multimodality treatment for locally unresectable T4 rectal cancer: a pooled analysis of the mayo clinic Rochester and Catharina hospital Eindhoven. J Gastrointest Oncol.

[11] Kusters M, Valentini V, Calvo FA (2010). Results of European pooled analysis of IORT-containing multimodality treatment for locally advanced rectal cancer: adjuvant chemotherapy prevents local recurrence rather than distant metastases. Ann Oncol.

[12] Mirnezami R, Chang GJ, Das P (2013). Intraoperative radiotherapy in colorectal cancer: systematic review and meta-analysis of techniques, long-term outcomes, and complications. Surg Oncol.

[13] Paunesku T, Woloschak GE (2017). Future directions of intraoperative radiation therapy: a brief review. Front Oncol.

[14] Petersen IA, Haddock MG, Donohue JH (2002). Use of intraoperative electron beam radiotherapy in the management of retroperitoneal soft tissue sarcomas. Int J Radiat Oncol Biol Phys.

[15] Pilar A, Gupta M, Ghosh Laskar S (2017). Intraoperative radiotherapy: review of techniques and results. Ecancermedicalscience.

[16] Qin HL, Lin CH, Zhang XL (2006). Evaluation of intraoperative radiotherapy for gastric carcinoma with D2 and D3 surgical resection. World J. Gastroenterol..

[17] Roeder F, Alldinger I, Uhl M (2018). Intraoperative electron radiation therapy in retroperitoneal sarcoma. Int J Radiat Oncol Biol Phys.

[18] Roeder F, Goetz JM, Habl G (2012). Intraoperative electron radiation therapy (IOERT) in the management of locally recurrent rectal cancer. BMC Cancer.

[19] Roeder F, Schulz-Ertner D, Nikoghosyan AV (2012). A clinical phase I/II trial to investigate preoperative dose-escalated intensity-modulated radiation therapy (IMRT) and intraoperative radiation therapy (IORT) in patients with retroperitoneal soft tissue sarcoma. BMC Cancer.

[20] Roeder F, Treiber M, Oertel S (2007). Patterns of failure and local control after intraoperative electron boost radiotherapy to the presacral space in combination with total mesorectal excision in patients with locally advanced rectal cancer. Int J Radiat Oncol Biol Phys.

[21] Skandarajah AR, Lynch AC, Mackay JR (2009). The role of intraoperative radiotherapy in solid tumors. Ann Surg Oncol.

[22] Skoropad VY, Berdov BA, Mardynski YS (2000). A prospective, randomized trial of pre-operative and intraoperative radiotherapy versus surgery alone in resectable gastric cancer. Eur J Surg Oncol.

[23] Wiig JN, Tveit KM, Poulsen JP (2002). Preoperative irradiation and surgery for recurrent rectal cancer. Will intraoperative radiotherapy (IORT) be of additional benefit? A prospective study. Radiother Oncol.

[24] Yu WW, Guo YM, Zhang Q (2015). Benefits from adjuvant intraoperative radiotherapy treatment for gastric cancer: a meta-analysis. Mol Clin Oncol.

[25] Zhang Q, Tey J, Peng L (2012). Adjuvant chemoradiotherapy with or without intraoperative radiotherapy for the treatment of resectable locally advanced gastric adenocarcinoma. Radiother Oncol.

